# Magnesium-enriched microenvironment promotes odontogenic differentiation in human dental pulp stem cells by activating ERK/BMP2/Smads signaling

**DOI:** 10.1186/s13287-019-1493-5

**Published:** 2019-12-10

**Authors:** Yuanyuan Kong, Xiaoli Hu, Yingqun Zhong, Ke Xu, Buling Wu, Jianmao Zheng

**Affiliations:** 1 Department of Stomatology, Nanfang Hospital, College of Stomatology, Southern Medical University, Guangzhou, 510055 China; 2 Key Laboratory of Oral Medicine, Guangzhou Institute of Oral Disease, Department of Endodontics, Stomatology Hospital of Guangzhou Medical University, Guangzhou, Guangdong China; 3 Guangdong Provincial Key Laboratory of Stomatology, Sun Yat-sen University, Guangzhou, Guangdong China; 4 Department of Operative Dentistry and Endodontics, Guanghua School of Stomatology, Affiliated Stomatological Hospital, Sun Yat-sen University, Guangzhou, 510055 Guangdong China

**Keywords:** Magnesium-enriched microenvironment, ERK, BMP2, Odontogenic differentiation, DPSC

## Abstract

**Background:**

Magnesium (Mg^2+^)-enriched microenvironment promotes odontogenic differentiation in human dental pulp stem cells (DPSCs), but the regulatory mechanisms remain undefined. The aim of this work was to assess magnesium’s function in the above process and to explore the associated signaling pathway.

**Methods:**

DPSCs underwent culture in odontogenic medium with the addition of 0, 1, 5, or 10 mM MgCl_2_. Intracellular Mg^2+^ levels in DPSCs were evaluated flow cytometrically using Mag-Fluo-4-AM. Mg^2+^-entry was inhibited by TRPM7 inhibitor 2-aminoethoxydiphenyl borate (2-APB). RNA-Sequencing was carried out for assessing transcriptome alterations in DPSCs during odontogenic differentiation associated with high extracellular Mg^2+^. KEGG pathway analysis was performed to determine pathways related to the retrieved differentially expressed genes (DEGs). Immunoblot was performed for assessing magnesium’s role and exploring ERK/BMP2/Smads signaling.

**Results:**

Mg^2+^-enriched microenvironment promoted odontogenic differentiation in DPSCs via intracellular Mg^2+^ increase. Consistently, the positive effect of high extracellular Mg^2+^ on odontogenic differentiation in DPSCs was blocked by 2-APB, which reduced Mg^2+^ entry. RNA-sequencing identified 734 DEGs related to odontogenic differentiation in DPSCs in the presence of high extracellular Mg^2+^. These DEGs participated in many cascades such as MAPK and TGF-β pathways. Consistently, ERK and BMP2/Smads pathways were activated in DPSCs treated with high extracellular Mg^2+^. In agreement, ERK signaling inhibition by U0126 blunted the effect of high extracellular Mg^2+^ on mineralization and odontogenic differentiation in DPSCs. Interestingly, BMP2, BMPR1, and phosphorylated Smad1/5/9 were significantly decreased by U0126, indicating that BMP2/Smads acted as downstream of ERK.

**Conclusions:**

Mg^2+^-enriched microenvironment promotes odontogenic differentiation in DPSCs by activating ERK/BMP2/Smads signaling via intracellular Mg^2+^ increase. This study revealed that Mg^2+^-enriched microenvironment could be used as a new strategy for dental pulp regeneration.

## Introduction

Dental pulp regeneration may be a potential treatment for managing permanent teeth undergoing necrosis [1]. Regenerative endodontic therapy has been assayed with multiple mesenchymal stem cells (MSCs), growth factors, and biological materials [2, 3]. Magnesium-containing scaffolds are considered to be ideal biomaterials for such treatment, with the properties of releasing Mg^2+^ to enhance dentin regeneration in MSCs [4].

As reported, Mg^2+^ acts as an intracellular second messenger connecting cell-surface receptor induction and cytosolic effectors [5]. Mg^2+^ is the fourth most abundant cation in the human body and is critical for ATP-dependent phosphorylation of DNA, RNA, and enzymes, and the average Mg^2+^ concentration in dentin is about 1% (wt/wt) [6–8]. Mg^2+^ is involved in the bio-mineralization of bones and teeth and directly affects crystallization and pattern generation of the inorganic mineral phase [8–10]. Evidence indicates mutations of the Mg^2+^ transporters TRPM7 and CNNM4 result in deficient dentin mineralization, confirming Mg^2+^ involvement in tooth development [11, 12]. Animals fed low Mg^2+^ diets show deficient dentin and enamel mineralization [13]. Our previous study also demonstrates that the Mg^2+^ transporter Magt1 plays an important role in odontogenic differentiation of bone marrow MSCs by regulating intracellular Mg^2+^ [14]. Studies have found high extracellular Mg^2+^ and its transporter regulate odontogenic differentiation in human DPSCs, with involvement in dentin mineralization [4, 15].

However, the regulatory mechanisms of Mg^2+^ effects on odontogenic differentiation in DPSCs remain undefined. Till now, the altered transcriptome of DPSCs undergoing odontogenic differentiation induced by an Mg^2+^-enriched microenvironment has not been assessed; thus, we determine the alteration of transcriptome using RNA sequencing. Moreover, we aim to clarify the role of Mg^2+^-enriched microenvironment in odontogenic differentiation of DPSCs and determine the associated signaling pathways.

## Materials and methods

### DPSC isolation and identification

Studies involving patients had approval from the Ethics Committee of Sun Yat-Sen University. DPSCs were collected from non-diseased pulp tissues of caries-free third molars of patients. DPSCs were isolated according to a previous report [16] and maintained in α-MEM containing 10% FBS (GIBCO, USA) and 10 mg/mL streptomycin and 10 U/mL penicillin (Sigma, USA). DPSCs at passages 3 to 7 were used in experiments. Flow cytometry analysis was performed to investigate DPSCs for surface markers. DPSCs were incubated with conjugated human antibodies, including CD45-PE, CD73-PE, CD90-APC, and CD166-PE (BD, USA), and assessed flow cytometrically (BD, USA). For confirming the adipogenic differentiation potential of stem cells, DPSCs underwent induction for 28 days with adipogenic medium containing 0.5 μM isobutyl-methylxanthine, 50 μM indomethacin, 0.5 μM dexamethasone, and 5 μg/mL insulin (Sigma, USA). To evaluate osteoblastic differentiation, DPSCs underwent culture for 14 days in osteogenic medium containing 100 nM dexamethasone, 10 mM β-glycerophosphate, and 0.2 mM ascorbic acid (Sigma, USA).

### Determination of DPSC proliferation

DPSCs seeded in 96-well plates at 1000 cells/well underwent culture in growth medium after addition of 0, 1, 5, or 10 mM MgCl_2_ for 1, 2, and 3 days, respectively. Cell Counting Kit-8 (Dojindo, Japan) was applied as directed by the manufacturer.

### Odontogenic differentiation of DPSCs induced by the Mg^2+^-enriched microenvironment

DPSCs seeded in 12-well plates at 3 × 10^4^ cells/well underwent culture in α-MEM containing 10% FBS at 37 °C. At 80% confluency, DPSC culture was performed in odontogenic medium with 100 nM dexamethasone, 10 mM β-glycerophosphate, and 0.2 mM ascorbic acid, with the addition of 0, 1, 5, or 10 mM MgCl_2_, respectively. After 7 days, the protein amounts of RUNX2, DSP, and DMP-1 were determined by automated immunoblot. After 14 days, mineralization nodules were assessed by Alizarin red S staining.

### Alizarin red S staining

Alizarin red S (Sigma, USA) staining was carried out as proposed by the manufacturer. Destaining was performed with 10% cetylpyridinium chloride monohydrate buffer for 30 min, and optical density was obtained at 575 nm. Relative extracellular matrix mineralization was calculated for each sample.

### Inhibition of Mg^2+^ entry by 2-aminoethoxydiphenyl borate (2-APB)

Given that TRPM7 was reported to transport Mg^2+^ across the cell membrane, we inhibited Mg^2+^-entry by treatment with 100 μM of the TRPM7 inhibitor 2-APB (Aladdin, China) [17].

### Automated immunoblot

Total protein from DPSCs was obtained with RIPA (Cell Signaling, USA). Automated immunoblot was carried out with Simple Wes (Protein Simple, USA) as directed by the manufacturer. In brief, 2.5 μg total protein samples were mixed with the standard fluorescent master mix and added to prefilled Wes assay plates, alongside antibody diluent (Protein Simple); primary antibodies targeting BMP2 (Affinity, USA), BMPR1 (Affinity), p-Smad1/5/9 (Affinity), RUNX2 (Affinity), DSP (Santa Cruz, USA), DMP-1 (Genetex, USA), ERK, p-ERK, JNK, p-JNK, p38, p-p38 (Cell Signaling), and β-Tublin (Affinity, USA) were probed; anti-rabbit and anti-mouse secondary antibodies conjugated to Streptavidin-HRP (Protein Simple) were used for detection. The compass software (Protein Simple) was utilized for analysis.

### Flow cytometry evaluation of intracellular magnesium

Intracellular magnesium amounts in DPSCs were assessed with Mag-Fluo-4-AM (Invitrogen, USA) as directed by the manufacturer. Briefly, Mag-Fluo-4-AM was added at 2 μM and incubated for 30 min. This was followed by three cell washes. Fluorescence intensities were obtained by flow cytometry (BD, USA) in triplicate assays.

### RNA sequencing (RNA-seq)

RNA-seq was carried out according to our previous work [14]. Briefly, DNase I treated total RNA specimens were enriched for mRNA using Oligo (dT) magnetic beads. Random-hexamer primers were employed for reverse transcription, and second-strand cDNA synthesis was carried out with Second Strand cDNA Synthesis Kit (Beyotime, China) as directed by the manufacturer. Short fragment purification used the QiaQuick PCR extraction kit (Qiagen, the Netherlands). Sequencing adapters were next added to these short fragments. Suitable fragments were amplified, and an Illumina HiSeq™ 2000 (Illumina Inc., USA) was employed for the cDNA sequencing. False discovery rate (FDR) adjusted *p* < 0.05 was obtained by the Benjamini-Hochberg method.

### GO and KEGG pathway analyses

Gene Ontology (GO, http://www.geneontology.org) and Kyoto Encyclopedia of Genes and Genomes pathway (KEGG, http://www.genome.jp/kegg/pathway.html) analyses were carried out for determining biological functions and enriched metabolic or signal transduction pathways associated with the differentially expressed genes (DEGs), respectively.

### MAPK signaling assessment

To assess MAPK signaling involvement, DPSCs underwent culture in 12-well plates in odontogenic medium with the addition of 0, 1, 5, or 10 mM MgCl_2_ for 7 days, respectively. Then, total protein from DPSCs was isolated to measure the protein amounts of ERK, p-ERK, JNK, p-JNK, p38, and p-p38 by automated immunoblot. During odontogenic differentiation of DPSCs administered 5 mM MgCl_2_ for 7 days, ERK/MAPK signaling was suppressed with 50 μM U0126 (AbMole BioScience, USA) for 2 h every 2 days.

### Statistical analysis

Experiments were performed three times. Data were expressed as mean ± SD and were assessed by ANOVA test with SPSS 17.0 (SPSS, USA); *p* < 0.05 indicated statistical significance.

## Results

### Effects of the Mg^2+^-enriched microenvironment on proliferation and differentiation in DPSCs

**Fig. 1:**
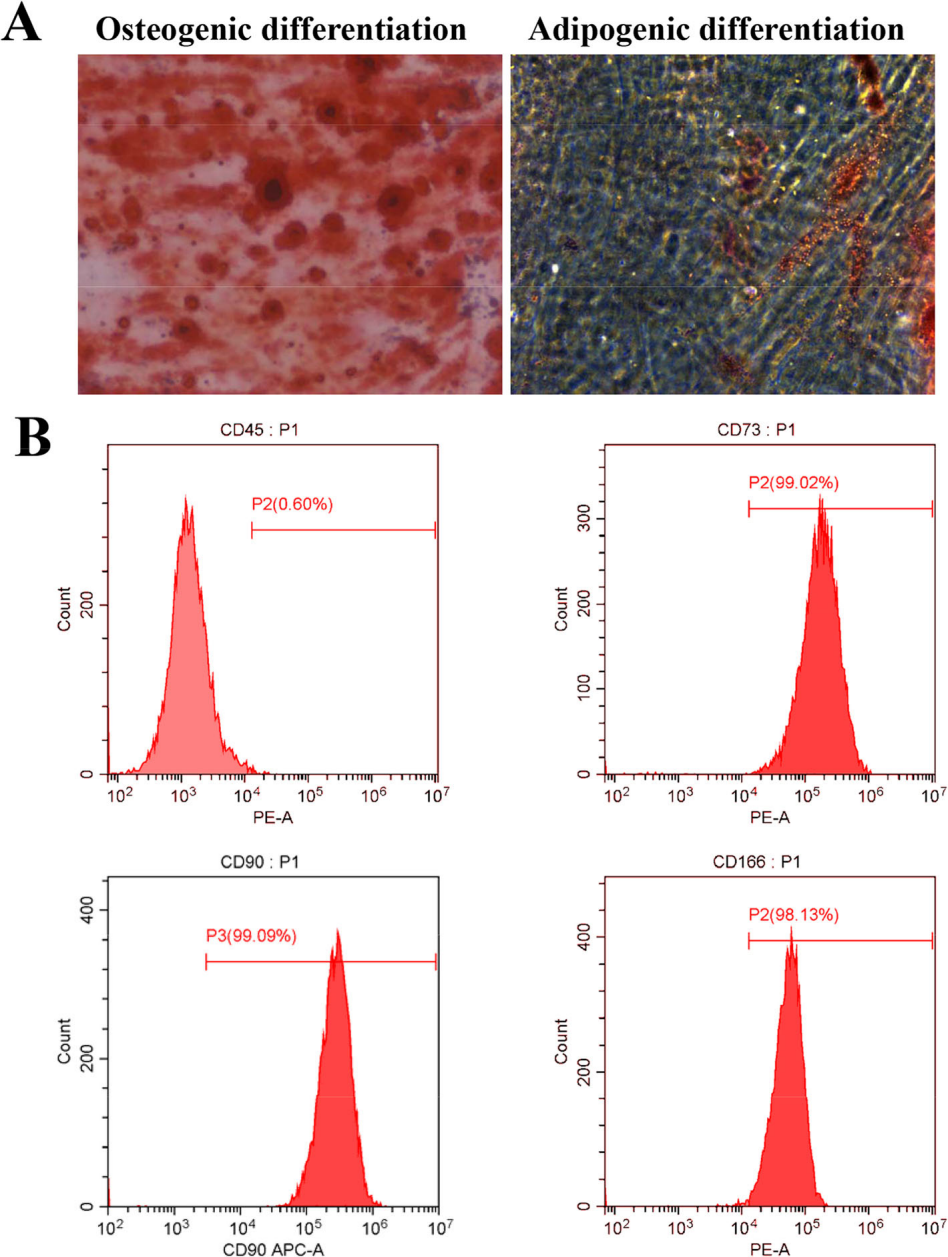
Identification of DPSCs. **a** Mineral deposits stained by Alizarin Red S in DPSCs following osteogenic differentiation for 2 weeks. Adipocyte staining by Oil Red O upon adipoinduction for 4 weeks. **b** The expression rates of CD45, CD73, CD90, and CD166 are shown

Passage 3 DPSCs were examined. As shown in Fig. 1a, DPSCs could differentiate into osteoblasts and adipocytes, suggesting their multi-lineage differentiation potential. Flow cytometry analysis showed DPSCs had low CD45 (hematopoietic cell marker) amounts (0.60%), but elevated amounts of the mesenchymal stem cell markers CD73 (99.02%), CD90 (99.09%), and CD166 (98.13%) (Fig. 1b).

**Fig. 2:**
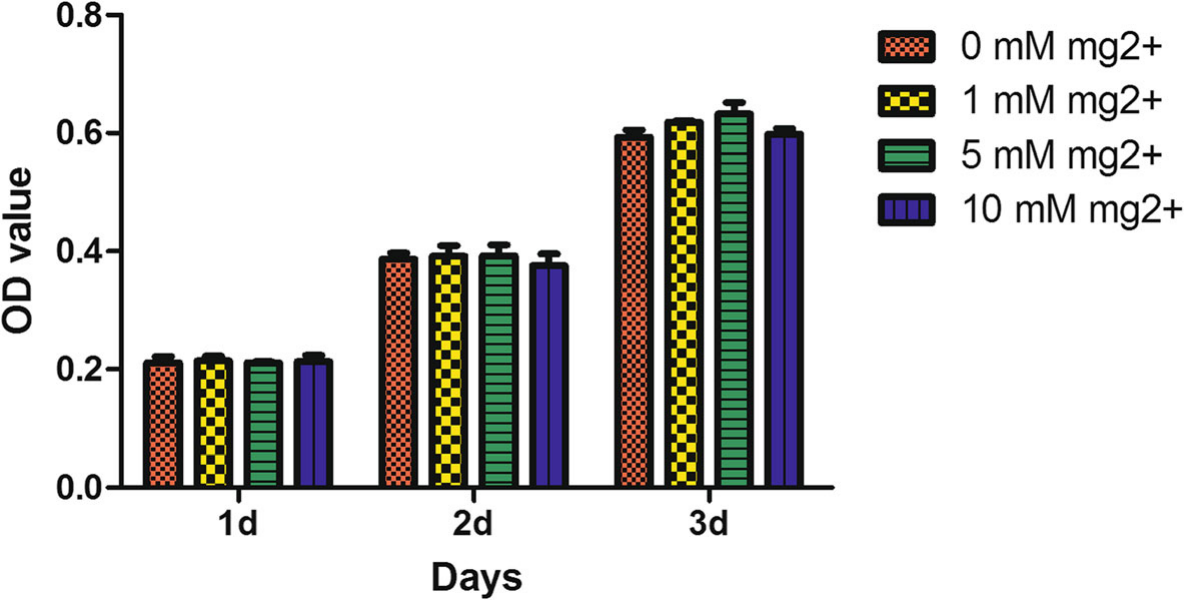
Cell Counting Kit-8 analysis showing that Mg^2+^-enriched microenvironment has no effect on DPSC proliferation

To evaluate the impact of high extracellular Mg^2+^ on their proliferation, DPSCs underwent incubation in Mg^2+^-enriched media at different concentrations (0 mM, 1 mM, 5 mM, and 10 mM) for 1, 2, and 3 days, respectively. Cell Counting Kit-8 analysis showed that high extracellular Mg^2+^ did not affect DPSC proliferation **(**Fig. 2**)**.

**Fig. 3:**
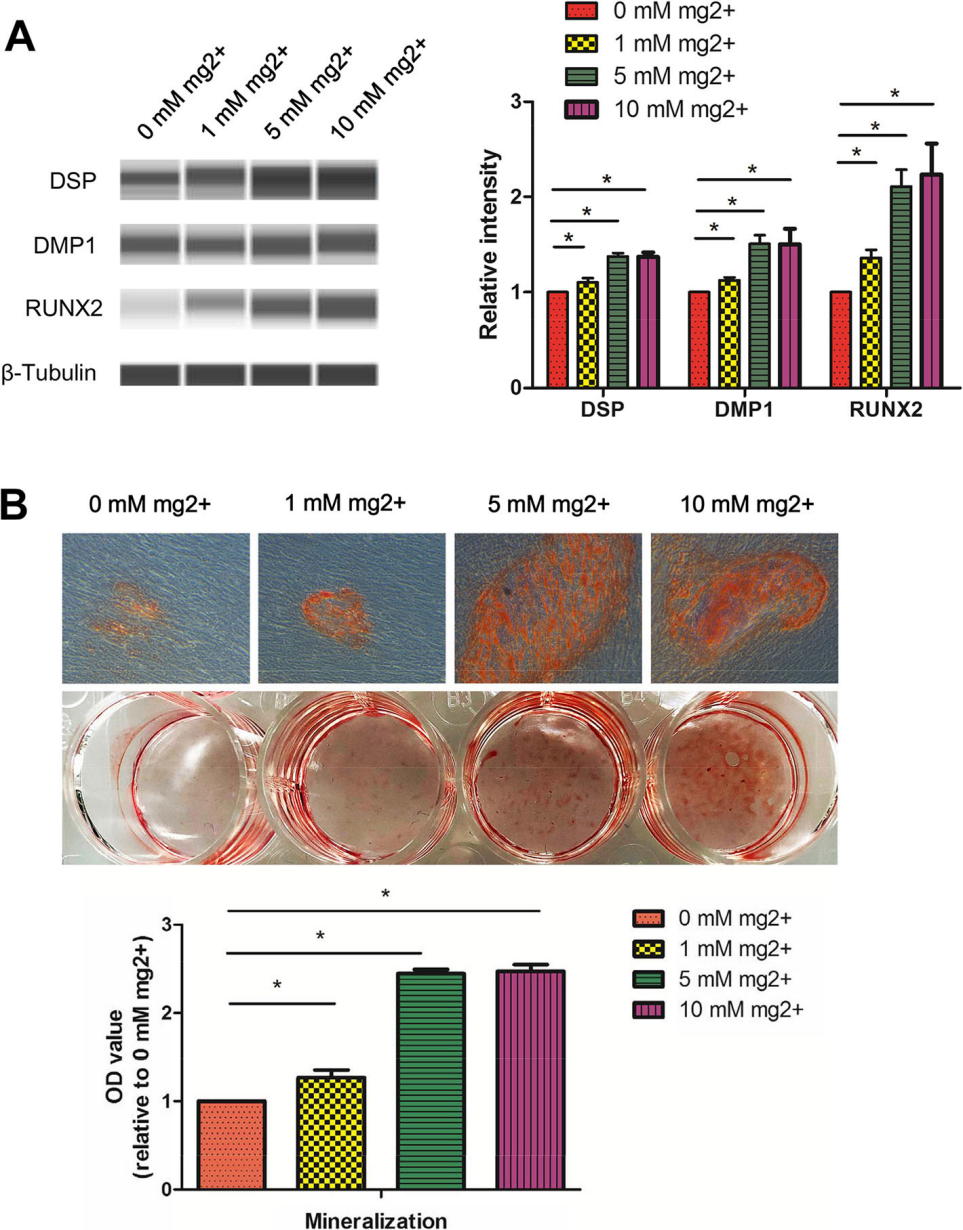
Effects of Mg^2+^-enriched microenvironment on DPSC differentiation. **a** After 7 days, RUNX2, DMP-1, and DSP protein amounts were markedly elevated in DPSCs treated with 1 mM, 5 mM, and 10 mM Mg^2+^, compared with the 0 mM Mg^2+^ group. **b** Consistently, mineralization nodules were also markedly increased after 14 days. However, there was no difference between the 5 and 10 mM Mg^2+^ groups

To analyze the impact of high extracellular Mg^2+^ on their differentiation, DPSCs were cultured in Mg^2+^-enriched odontogenic medium at different concentrations (0 mM, 1 mM, 5 mM, and 10 mM). After 7 days, RUNX2, DMP-1, and DSP protein amounts were markedly elevated in DPSCs treated with 1 mM, 5 mM, and 10 mM Mg^2+^, respectively, compared with 0 mM Mg^2+^ (Fig. 3a). Consistently, mineralization nodules were also markedly increased (Fig. 3b). However, there was no difference between the 5 and 10 mM Mg^2+^ groups **(**Fig. 3**)**. These results showed that the Mg^2+^-enriched microenvironment promoted the odontogenic differentiation of DPSCs.

### Mg^2+^-enriched microenvironment promotes odontogenesis in DPSCs by increasing intracellular Mg^2+^

**Fig. 4:**
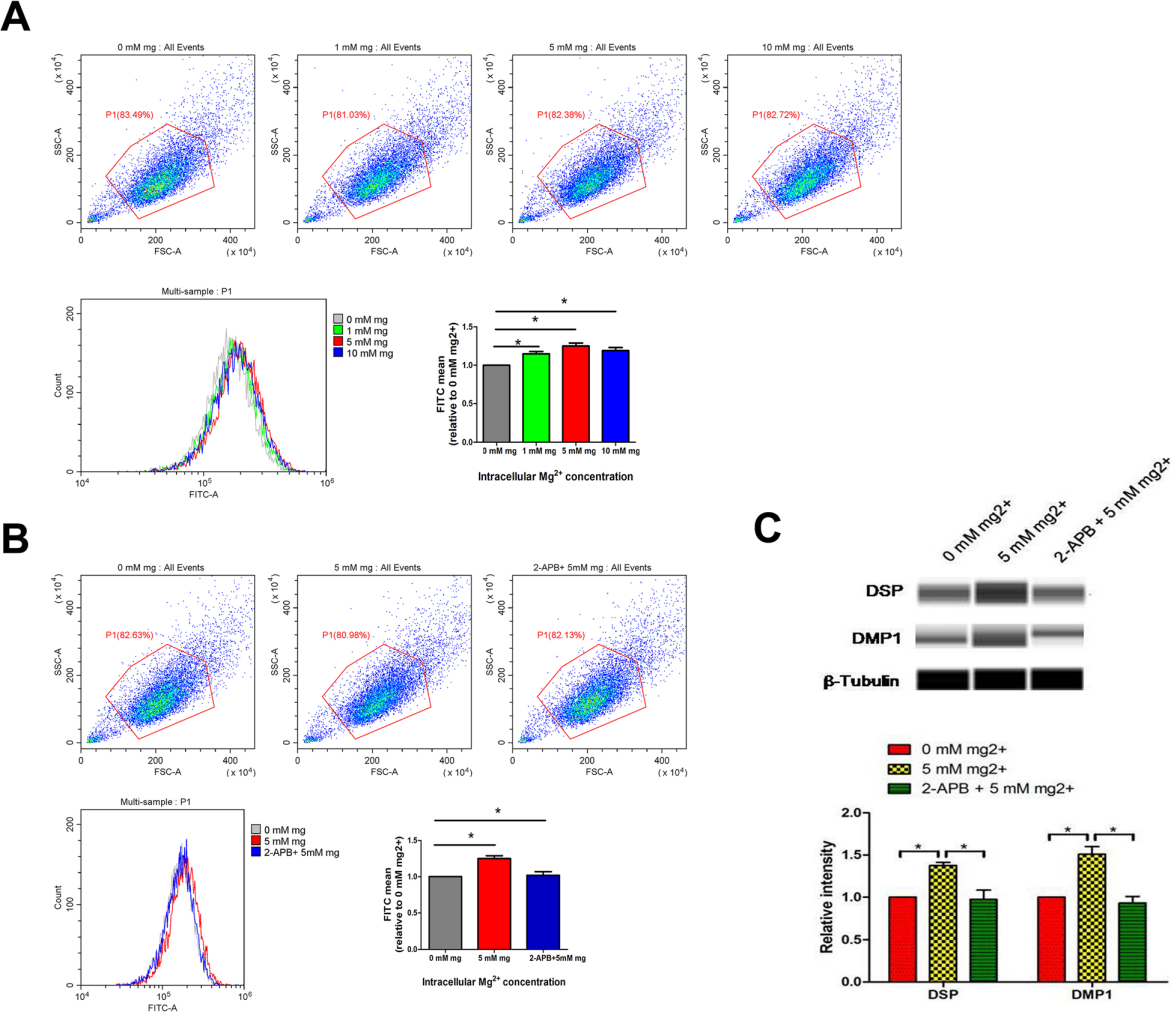
Mg^2+^-enriched microenvironment promotes odontogenic differentiation in DPSCs by increasing intracellular Mg^2+^. **a** Intracellular Mg^2+^ was significantly increased in DPSCs exposed to high extracellular Mg^2+^ as assessed flow cytometrically using Mag-Fluo-4-AM; however, there was no difference between the 5 and 10 mM Mg^2+^ groups. **b** Mg^2+^-entry was significantly inhibited by the TRPM7 inhibitor 2-APB. **c** The positive effect of Mg^2+^-enriched microenvironment on odontogenic differentiation in DPSCs was also blocked by 2-APB

Intracellular magnesium levels in DPSCs treated with Mg^2+^-enriched odontogenic media at different concentrations (0 mM, 1 mM, 5 mM, and 10 mM) were assessed flow cytometrically using Mag-Fluo-4-AM. Figure 4a suggests that intracellular Mg^2+^ was starkly increased in DPSCs treated with 1 mM, 5 mM, and 10 mM Mg^2+^, compared with the 0 mM Mg^2+^ group. However, there was no difference in intracellular magnesium of DPSCs treated with 5 mM and 10 mM Mg^2+^, which is consistent with their comparable effects on odontogenic differentiation.

Given that TRPM7 was reported to transport Mg^2+^ across the cell membrane [17], we next examined the effects of its blocker 2-APB. The results showed that Mg^2+^ entry was significantly blunted by 2-APB (100 μM, inhibiting TRPM7 [17]) **(**Fig. 4b**).** The positive effect of high extracellular Mg^2+^ (5 mM Mg^2+^) on odontogenic differentiation of DPSCs was also blocked by 2-APB **(**Fig. 4c**)**. These results indicated that high extracellular Mg^2+^ promoted odontogenic differentiation in DPSCs via intracellular Mg^2+^ increase.

### High extracellular Mg^2+^ alters the transcriptome of DPSCs

**Fig. 5:**
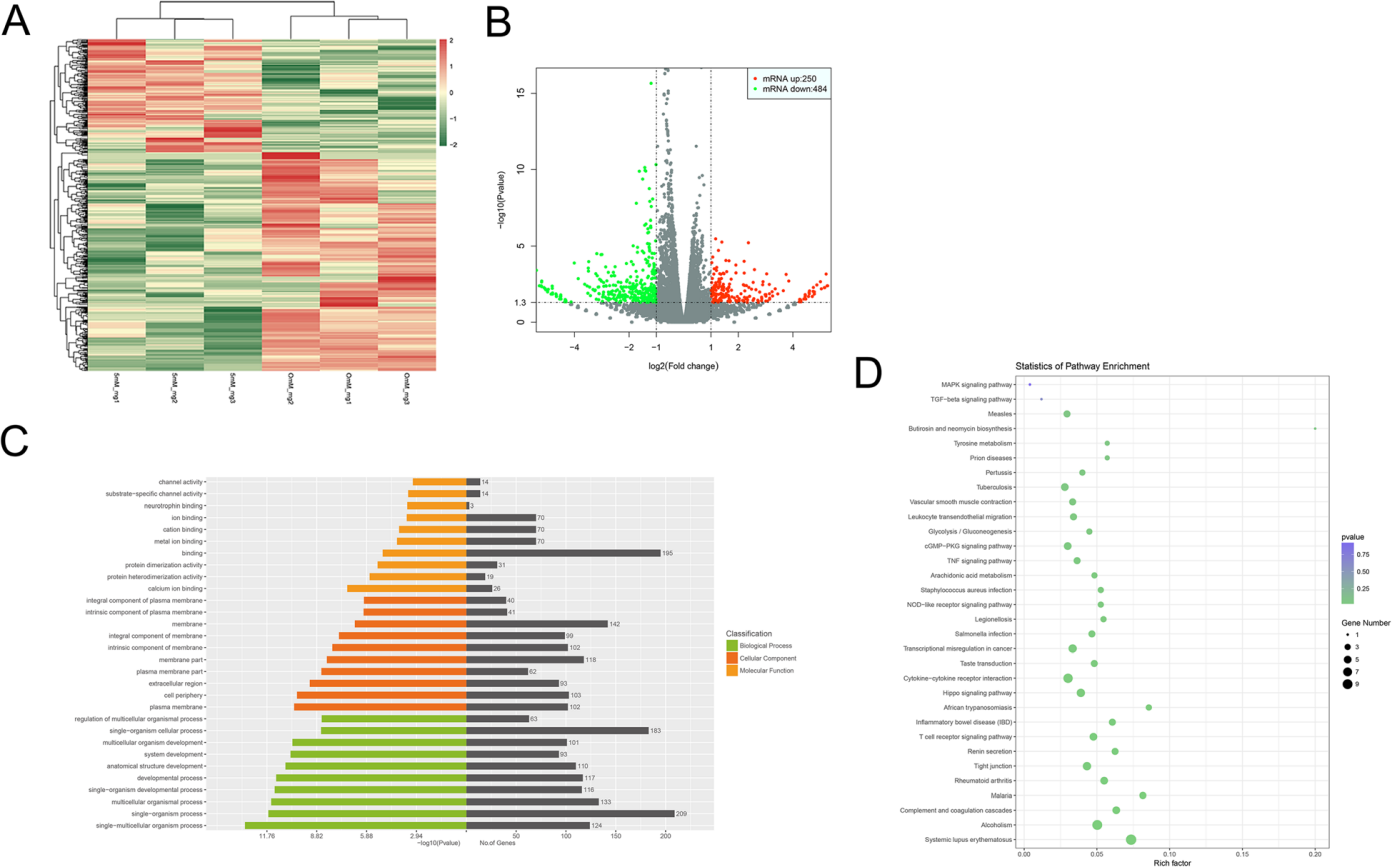
RNA-Seq of DPSCs undergoing odontogenic differentiation upon induction by high extracellular Mg^2+^. **a**, **b** There were734 genes significantly altered, with 250 upregulated and 484 downregulated. **c** GO analysis of differentially expressed genes (DEGs). **d** KEGG pathway analysis revealed that the DEGs were involved in many signaling pathways, including MAPK and TGF-β pathways

Compared with control cells (0 mM Mg^2+^), exposure to high extracellular Mg^2+^ (5 mM Mg^2+^) for 7 days resulted in 734 DEGs in DPSCs, including 250 upregulated and 484 downregulated **(**Fig. 5a, b**)**.

### GO and KEGG pathway analyses of DEGs

In GO analysis, DEGS were mostly involved in developmental process, anatomical structure development, organism development, system development, channel activity, metal ion binding, integral component of plasma membrane, etc. **(**Fig. 5c**)**. KEGG pathway analysis indicated that the DEGs retrieved participated in many pathways, e.g., MAPK and TGF-β pathways **(**Fig. 5d**)**.

### ERK signaling is activated by high extracellular Mg^2+^ during odontogenic differentiation of DPSCs

**Fig. 6:**
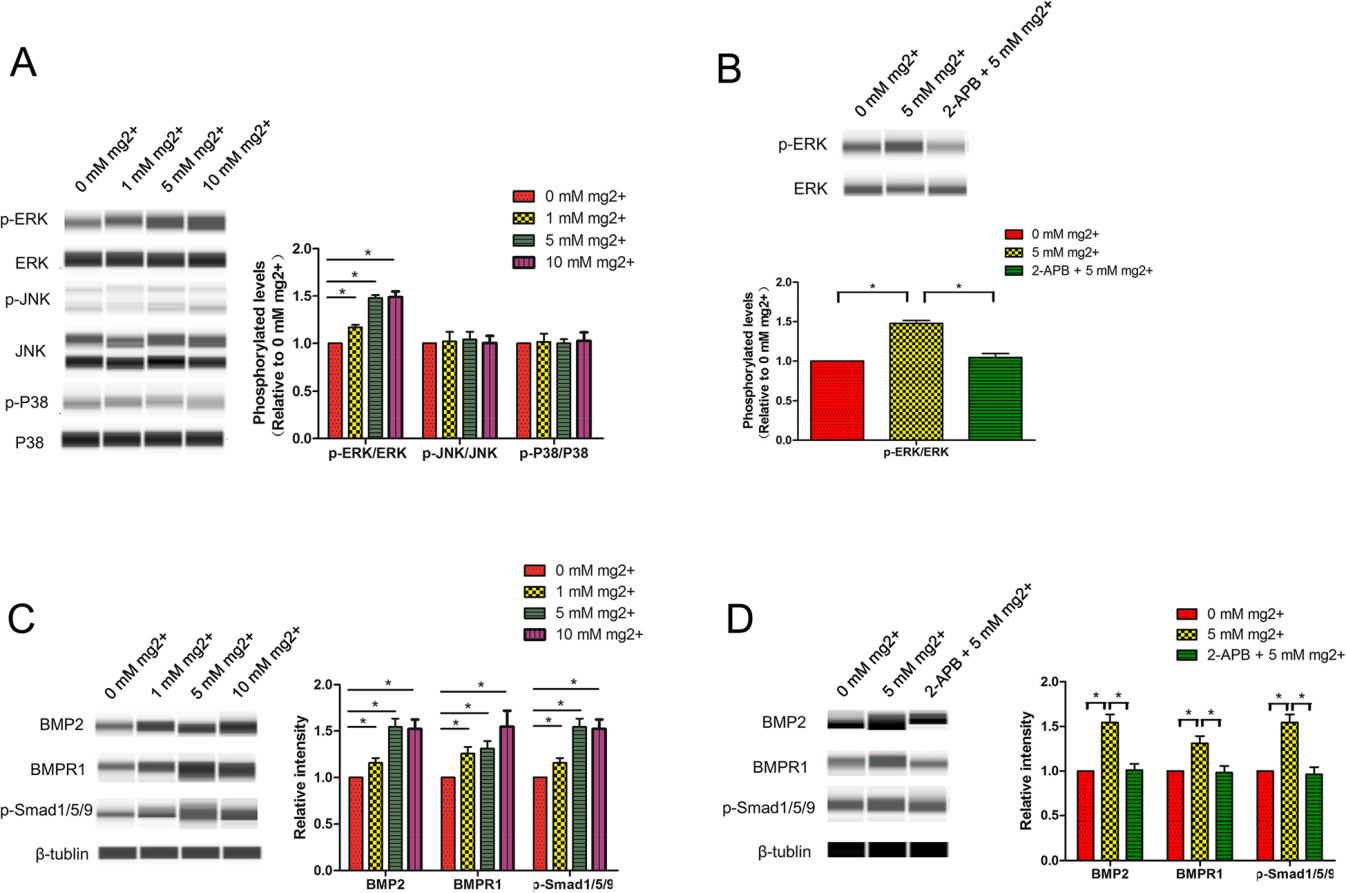
The ERK and BMP2 signaling pathway is activated by high extracellular Mg^2+^ in DPSCs during odontogenic differentiation. **a** ERK phosphorylation was significantly enhanced in DPSCs treated with 1 mM, 5 mM, and 10 mM Mg^2+^ compared with the 0 mM Mg^2+^ group, but p38 and JNK phosphorylation amounts were unchanged. **b** ERK phosphorylation was reduced by 2-APB. **c** Consistently, the protein levels of BMP2, BMPR1, and phosphorylated Smad1/5/9 were significantly increased in DPSCs exposed to high extracellular Mg^2+^. **d** BMP2, BMPR1, and phosphorylated Smad1/5/9 protein amounts were decreased by 2-APB

As shown above, KEGG pathway analysis revealed MAPK signaling activation by high extracellular Mg^2+^during odontogenic differentiation of DPSCs. To verify this finding, DPSCs were incubated in odontogenic medium with the addition of 0, 1, 5, and 10 mM MgCl_2_ for 7 days, respectively. The results showed that ERK phosphorylation was markedly enhanced in DPSCs treated with 1 mM, 5 mM, and 10 mM Mg^2+^ compared with the 0 mM Mg^2+^ group, but p38 and JNK phosphorylation showed no change **(**Fig. 6a**)**. In accordance, the positive effect of high extracellular Mg^2+^ on ERK phosphorylation was blocked by 2-APB (100 μM, inhibiting TRPM7) **(**Fig. 6b**)**, which reduced intracellular Mg^2+^amounts **(**Fig. 4b**)**.

### BMP2/Smads signaling is activated by high extracellular Mg^2+^during odontogenic differentiation of DPSCs

As reported previously, BMP2 is a TGF-β family member controlling odontogenic differentiation of stem cells [18, 19]. To verify the above KEGG pathway data, we performed Western blot to determine whether BMP2/Smads signaling is activated by high extracellular Mg^2+^. The results showed that BMP2, BMPR1, and phosphorylated Smad1/5/9 were significantly increased in DPSCs cultured in odontogenic medium containing high extracellular Mg^2+^**(**Fig. 6c**)**. In accordance, the positive effects of high extracellular Mg^2+^ on BMP2, BMPR1, and phosphorylated Smad1/5/9 were blocked by 2-APB (100 μM, inhibiting TRPM7) **(**Fig. 6d**)**.

### Mg^2+^-enriched microenvironment promotes odontogenic differentiation in DPSCs via ERK/BMP2/Smads signaling

**Fig. 7:**
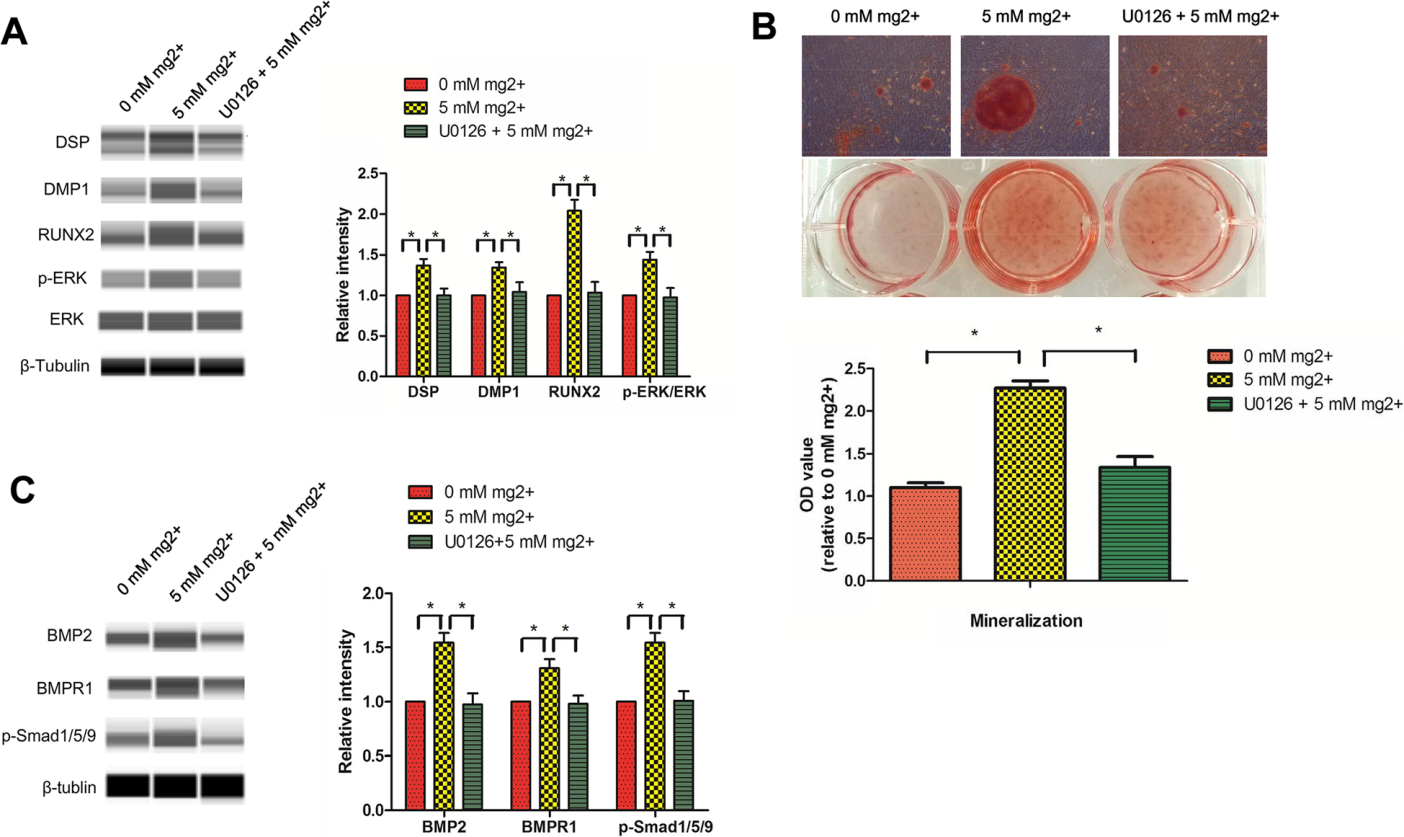
Mg^2+^-enriched microenvironment promotes odontogenic differentiation in DPSCs via ERK/BMP2/Smads signaling. **a** After treatment with U0126, the protein amounts of RUNX2, DSP, and DMP-1 were markedly decreased in DPSCs treated with high extracellular Mg^2+^. **b** Consistently, mineralization nodules were also reduced. **c** After treatment with U0126, BMP2, BMPR1, and phosphorylated Smad1/5/9 protein amounts were significantly decreased, indicating that BMP2/Smads signaling acted as downstream of ERK

We inhibited ERK signaling with U0126, and mineralization nodules were markedly decreased, as well as RUNX2, DSP, and DMP-1 protein amounts in DPSCs treated with Mg^2+^-enriched odontogenic medium (5 mM Mg^2+^) for 7 days **(**Fig. 7a,b**)**. Interestingly, BMP2, BMPR1, and phosphorylated Smad1/5/9 amounts were also significantly decreased by U0126, indicating that BMP2/Smads acted as downstream of ERK **(**Fig. 7c**)**.

**Fig. 8:**
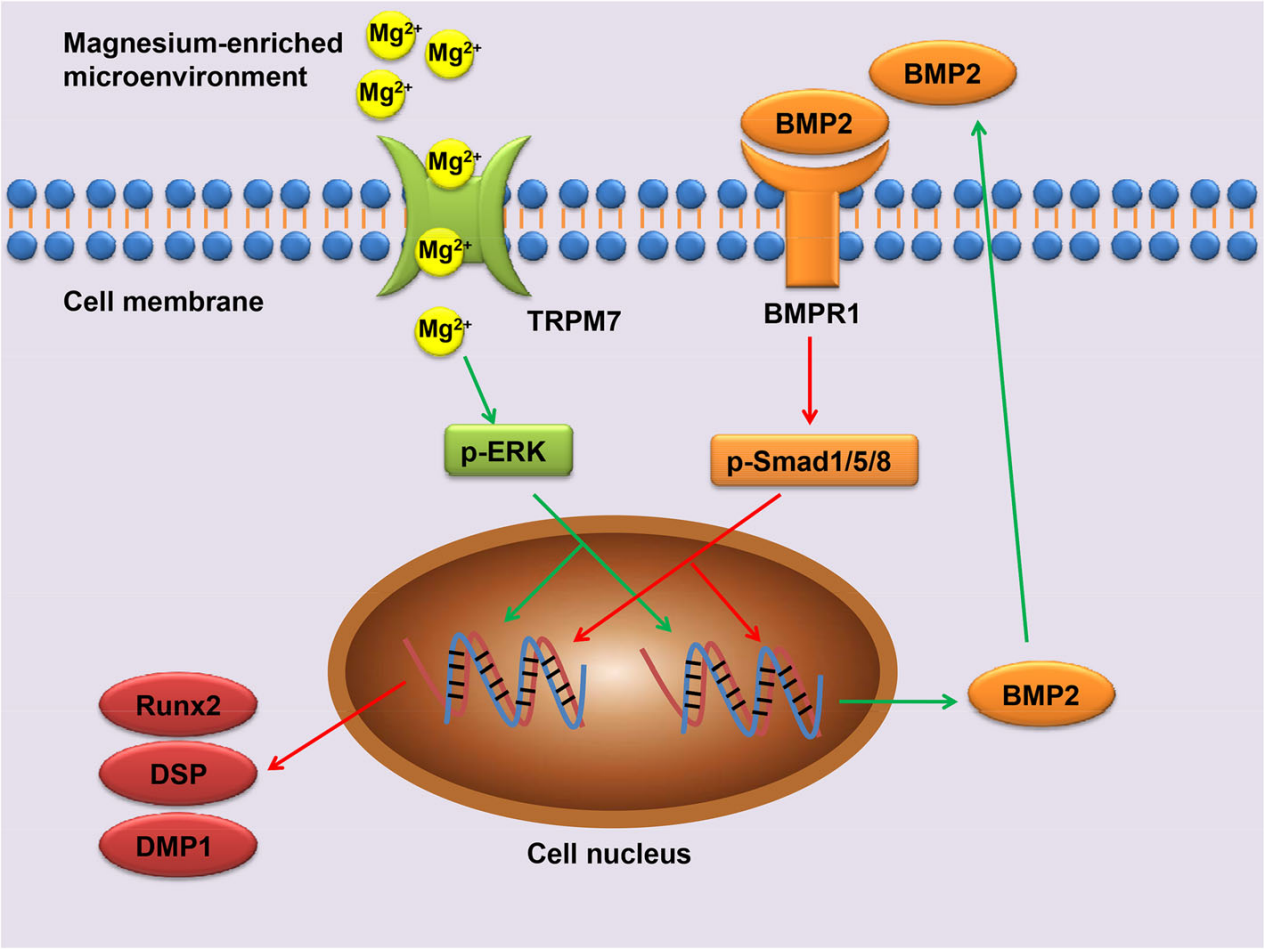
Summary diagram. Mg^2+^-enriched microenvironment promotes odontogenic differentiation in DPSCs by activating ERK/BMP2/Smads signaling via intracellular Mg^2+^ increase

Taken together, these findings suggested that Mg^2+^-enriched microenvironment promoted odontogenic differentiation in DPSCs by activating ERK/BMP2/Smads signaling via intracellular Mg^2+^ increase **(**Fig. 8**)**.

## Discussion

Regenerative endodontic therapy involves biological procedures for replacing damaged dental structures such as dentin and root, and cells of the dentin/pulp complex [20]. DPSCs are suitable for such treatment, and their differentiation into odontogenic stem cells associated with biomaterials and growth factors is critical for dental pulp regeneration [1]. It is known that magnesium-containing scaffolds are ideal tools for regenerative endodontic therapy, releasing high extracellular Mg^2+^ to enhance dentin regeneration in DPSCs [4]. Our previous studies found the Mg^2+^ transporters TRPM7 and MagT1 regulate odontogenic differentiation in stem cells [14, 15]. Nakano and collaborators [12] reported that TRPM7 silencing in mice results in marked tooth hypomineralization, suggesting TRPM7 to be involved in enamel and dentin biomineralization by supplying enough Mg^2+^ for ALPL activity. Luder and colleagues [11] demonstrated that mutation of CNNM4, a Mg^2+^ transporter, caused mineralization abnormalities in both enamel and dentin, in association with starkly reduced magnesium amounts. Consistent with previous studies, the present work determined that Mg^2+^-enriched microenvironment enhanced odontogenic differentiation in DPSCs by altering the formation of mineralization nodules as well as RUNX2, DMP-1, and DSP protein amounts, through intracellular Mg^2+^ concentration increase.

However, how high extracellular Mg^2+^ affects odontogenic differentiation remains unknown. Therefore, RNA-Seq was carried out to assess transcriptome alterations in DPSCs exposed to Mg^2+^-enriched microenvironment. Interestingly, GO and KEGG pathway analyses revealed 734 DEGs contributing to odontogenic differentiation in DPSCs induced by high extracellular Mg^2+^. The obtained DEGs participated in many pathways, such as MAPK and TGF-β pathways.

MAPK signaling regulates stem cell proliferation, migration, and differentiation [21–23]. Yu and colleagues [24] demonstrated that dentine matrix promoted odontogenic differentiation in bone marrow MSCs by inducing ERK and P38 MAPK pathways. Huang and collaborators [25] revealed that dental pulp cell-derived exosomes promote odontogenic differentiation in bone marrow MSCs via P38/MAPK signaling. As reported in previous studies, MAPK signaling also contributes to odontogenic differentiation in DPSCs [26]. In the present study, ERK phosphorylation was significantly increased in DPSCs treated with high extracellular Mg^2+^, but p38 and JNK phosphorylation levels remained unchanged, indicating ERK/MAPK signaling is induced by high extracellular Mg^2+^. Consistently, blunting ERK/MAPK signaling with U0126 blocked the effects of high extracellular Mg^2+^ on mineralization and odontogenic differentiation in DPSCs.

According to previous studies, intracellular Mg^2+^ plays a pivotal role in ERK/MAPK signaling induction [27–30]. For example, Mg^2+^ deprivation downregulates p-ERK1/2 in MDCK cells, and Mg^2+^ re-supplementation reverses this effect [29, 30]. Meanwhile, TRPM7 suppression inhibits ERK phosphorylation by reducing intracellular Mg^2+^ amounts [27, 28]. Our previous studies also revealed that suppression of MagT1 inactivates the ERK/MAPK pathway by decreasing intracellular Mg^2+^, which results in inhibited odontogenic differentiation of bone marrow MSCs [14]. In the present study, intracellular magnesium in DPSCs exposed to high extracellular Mg^2+^ was assessed flow cytometrically with Mag-Fluo-4-AM. As shown above, intracellular Mg^2+^ amounts were starkly increased in DPSCs treated with high extracellular Mg^2+^. In accordance, the positive effect of high extracellular Mg^2+^ on ERK phosphorylation was blocked by 2-APB, which reduces intracellular Mg^2+^ amounts, indicating that high extracellular Mg^2+^ activates ERK/MAPK signaling by increasing intracellular Mg^2+^.

BMP2, a member of the TGF-β family of proteins, contributes to odontogenic differentiation in DPSCs [18, 19]. Consistent with KEGG pathway data, BMP2, BMPR1, and phosphorylated Smad1/5/9 amounts were significantly increased in DPSCs incubated in Mg^2+^-enriched odontogenic media. As expected, the positive effects of high extracellular Mg^2+^ on BMP2, BMPR1, and phosphorylated Smad1/5/9 were blocked by the Mg^2+^transporter inhibitor 2-APB, indicating that BMP2/Smads signaling is activated during odontogenic differentiation induced by Mg^2+^-enriched microenvironment. This finding corroborated Cheng et al. [31] showing that high-purity magnesium scaffolds promote bone regeneration in rabbits via activation of BMP2 signaling by releasing high amounts of extracellular Mg^2+^.

Interestingly, ERK signaling inhibition by U0126 resulted in significantly decreased BMP2, BMPR1, and phosphorylated Smad1/5/9 amounts, indicating that BMP2/Smads is a downstream signaling of ERK. Taken together, these findings demonstrated that high extracellular Mg^2+^ promotes odontogenic differentiation in DPSCs by activating ERK/BMP2/Smads signaling via intracellular Mg^2+^increase.

## Conclusions

Overall, significant transcriptome alterations were detected in DPSCs during odontogenic differentiation associated with high extracellular Mg^2+^. In addition, Mg^2+^-enriched microenvironment enhanced odontogenic differentiation in DPSCs by activating ERK/BMP2/Smads signaling via intracellular Mg^2+^ increase.

## Data Availability

The authors confirm the availability of all data generated or analyzed in this manuscript.

## Abbreviations

RNA-Seq: RNA-Sequencing
MAPK: Mitogen-activated protein kinase
ERK: Extracellular signal-related kinase
JNK: c-Jun NH2-terminal kinase
DSP: Dentin sialophosphoprotein
DMP-1: Dentin matrix protein 1
GO: Gene Ontology
RUNX2: Runt-related transcription factor 2
BMP2: Bone morphogenetic protein 2
BMPR1: Bone morphogenetic protein receptor 1
